# Structures of substrate- and product-bound forms of a multi-domain copper nitrite reductase shed light on the role of domain tethering in protein complexes

**DOI:** 10.1107/S2052252520005230

**Published:** 2020-04-25

**Authors:** Daisuke Sasaki, Tatiana F. Watanabe, Robert R. Eady, Richard C. Garratt, Svetlana V. Antonyuk, S. Samar Hasnain

**Affiliations:** a Molecular Biophysics Group, Institute of Systems, Molecular and Integrative Biology, Faculty of Health and Life Sciences, University of Liverpool, Liverpool L69 7ZB, United Kingdom; b The São Carlos Institute of Physics, University of São Paulo, São Carlos 13563-120, Brazil

**Keywords:** nitro­gen cycle, denitrification, copper-containing nitrite reductase, electron transfer, catalysis, structural biology

## Abstract

Copper-containing nitrite reductases (CuNiRs) are found in all three kingdoms of life and play a major role in the denitrification branch of the global nitro­gen cycle where nitrate is used in place of di­oxy­gen as an electron acceptor in respiratory energy metabolism. Several C- and N-terminal redox domain tethered CuNiRs have been identified and structurally characterized during the last decade. Our understanding of the role of tethered domains in these new classes of three-domain CuNiRs, where an extra cytochrome or cupredoxin domain is tethered to the catalytic two-domain CuNiRs, has remained limited. This is further compounded by a complete lack of substrate-bound structures for these tethered CuNiRs. There is still no substrate-bound structure for any of the as-isolated wild-type tethered enzymes. Here, structures of nitrite and product-bound states from a nitrite-soaked crystal of the N-terminal cupredoxin-tethered enzyme from the *Hyphomicrobium denitrificans* strain 1NES1 (*Hd*_1NES1_NiR) are provided. These, together with the as-isolated structure of the same species, provide clear evidence for the role of the N-terminal peptide bearing the conserved His27 in water-mediated anchoring of the substrate at the catalytic T2Cu site. Our data indicate a more complex role of tethering than the intuitive advantage for a partner-protein electron-transfer complex by narrowing the conformational search in such a combined system.

## Abstract 2

Tethered protein–protein complexes are presumed to offer functional advantages such as electron transfer due to constraints imposed on the conformational search. This work shows that tethering has a much more complex role.

## 1. Introduction   

Denitrification is an important process in the global nitro­gen cycle and has significant impacts on agronomy, the environment and health (Zumft, 1997 ▸). Copper-containing nitrite reductases (CuNiRs) are found in all three kingdoms of life and catalyze the reduction of nitrite to nitric oxide, which is the first committed step of denitrification; NO_2_^−^ + e^−^ + 2 H^+^ 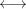 NO + H_2_O. The homotrimer structures are highly conserved among all the two-domain CuNiRs from organisms involved in agricultural denitrification such as *Alcaligenes xylosoxidans* (*Ax*) and *Achromobacter cyclo­clastes* (*Ac*) to bacterial pathogens *Neisseria gonorrhoeae* (*Ng*) and *Neisseria meningitides* (*Nm*). The catalytic type-2 copper center (T2Cu) is located at the interface of the adjacent monomers and is ‘hard-wired’ to the electron-donating type-1 copper center (T1Cu) via neighboring residues that form a Cys–His electron-transfer (ET) bridge. The T1Cu is close to the protein surface and functions as the electron acceptor from the physiological electron donor, cytochrome (Nojiri *et al.*, 2009 ▸) or pseudo­azurin (Nojiri, 2016 ▸). The two active-site residues, Asp_CAT_ and His_CAT_ around the T2Cu, are involved in substrate binding and catalysis with both residues starting in the deprotonated state prior to substrate-binding events (Godden *et al.*, 1991 ▸; Dodd *et al.*, 1998 ▸; Antonyuk *et al.*, 2005 ▸; Boulanger *et al.*, 2000 ▸; Tocheva *et al.*, 2004 ▸; Kataoka *et al.*, 2000 ▸; Fukuda *et al.*, 2016 ▸; Halsted *et al.*, 2019 ▸).

More recently, new classes of three-domain CuNiRs have been structurally characterized, where an extra cytochrome (Antonyuk *et al.*, 2013 ▸; Tsuda *et al.*, 2013 ▸) or cupredoxin (Opperman *et al.*, 2019 ▸) domain is tethered to the C-terminus of the catalytic core domain corresponding to the two-domain CuNiR. Both of the C-terminal cytochrome-tethered CuNiRs from *Ralstonia pickettii* (*Rp*) (Antonyuk *et al.*, 2013 ▸) and from *Pseudoalteromonas haloplanktis* (*Ph*) (Tsuda *et al.*, 2013 ▸) show a trimeric structure but reveal different linking arrangements for the tethered cytochrome and T1Cu–T2Cu core domains *via* a long tethering linker. In both cases, however, these alternative arrangements place the heme of the cytochrome adjacent to the T1Cu at a distance of ∼10 Å for an effective ET from the heme to the T1Cu. The latest addition to the CuNiR family is the C-terminal cupredoxin-tethered CuNiR from *Thermus scotoductus* (*Ts*), the structure of which was very recently elucidated (Opperman *et al.*, 2019 ▸). This enzyme, *Ts*NiR, is trimeric with the T1Cu_C_ (T1Cu in the tethered cupredoxin domain) located near the core T1Cu with an ET compatible distance of ∼14 Å. In contrast to *Rp*NiR and *Ph*NiR, the tethered C-terminal domain interacts directly with the T1Cu–T2Cu core domain of the same subunit.

The only known structure of an N-terminal cupredoxin-tethered CuNiR is for the enzyme from *Hyphomicrobium denitrificans* strain A3151 (*Hd*_A3151_NiR) (Nojiri *et al.*, 2007 ▸). Surprisingly its structure showed a prism-shaped homohexamer, whose monomers are organized into a tightly associated dimer of trimers with additional N-terminal cupredoxin (T1Cu_N_) domains interacting head-to-head. Unlike the three representatives of the C-terminal tethered *Rp*NiR (cytochrome), *Ph*NiR (cytochrome) and *Ts*NiR (cupredoxin), the tethered T1Cu_N_ domain is located far from the T1Cu–T2Cu catalytic core with a distance of ∼24 Å between the T1Cu_N_ and the T1Cu, thus questioning the role of N-terminal tethering in ET and catalysis. Pulse-radiolysis data (Nojiri *et al.*, 2007 ▸) for *Hd*_A3151_NiR obtained in the presence of nitrite suggest that generated electrons attack the T1Cu_N_, but not the T1Cu_core_. Subsequently, the reduced T1Cu_N_ gives up an electron to the type-2 Cu through the T1Cu_core_.

Our understanding of the role of tethered domains has been seriously hampered by the lack of substrate/product-bound structures of any tethered CuNiRs. This scarcity of substrate/product-bound structures extends to the C-terminal tethered CuNiRs, with the exception of *Rp*NiR, where it required either mutation of the gate-keeper residue Tyr323 or alternatively Asp_CAT_ as well as pre-incubation of the crystals with nitric oxide (Dong *et al.*, 2018 ▸; Hedison *et al.*, 2019 ▸). Deconstruction of *Rp*NiR into the NiR catalytic core and cytochrome domain showed the linker region that connects the two and harbors the gatekeeper tyrosine which unravels and results in a substantial conformational movement of the tethered cytochrome domain. This may place it far away from the catalytic core (T1Cu–T2Cu) suggesting that, in tethered domains, conformational dynamics may play an important role in substrate binding and regulation of catalysis (Hedison *et al.*, 2019 ▸) in these tethered systems.

Here, we have identified, characterized and determined the crystallographic structure of the N-terminal cupredoxin tethered *Hd*NiR from an alternative strain 1NES1 (*Hd*_1NES1_NiR) and compared it with *Hd*_A3151_NiR. The chromatographic profile showed *Hd*_1NES1_NiR to be primarily a hexamer but with enzymatic activity significantly lower than the classic two-domain CuNiRs. *Hd*_1NES1_NiR crystallizes in a different space group, *P*6_5_22, compared with *P*4_1_ in the case of *Hd*_A3151_NiR, with a trimer of *Hd*_1NES1_NiR in the asymmetric unit. It also forms a hexameric structure resulting from a dimer of trimers with the T1Cu_N_ located again too far away from the catalytic T1Cu–T2Cu NiR core for an effective ET. Despite a high sequence identity of 84% between *Hd*_1NES1_NiR and *Hd*_A3151_NiR, significant structural differences were observed, which may account for more than an order of magnitude difference in specific NiR activity of these enzymes. Remarkably, in contrast to all other tethered CuNiR enzymes, we have been able to obtain a substrate-bound structure for *Hd*_1NES1_NiR by simply soaking crystals with nitrite in a manner similar to the classic two-domain CuNiRs. In fact, structure determination of one nitrite-soaked *Hd*_1NES1_NiR crystal revealed the trimeric assembly in the asymmetric unit with the catalytic T2Cu in both substrate- and product-bound states. In two molecules T2Cu bound the substrate at full occupancy and one molecule had NO bound to the T2Cu site, consistent with the functional asymmetry that has been noted recently for classic two-domain trimeric CuNiRs (Hedison *et al.*, 2019 ▸). This is the first clear structural evidence for such asymmetry, suggesting a one-third reactivity of the T2Cu center of the core enzyme.

## 2. Materials and methods   

### 2.1. Primary structure alignment   

Primary sequence alignment was performed with *ClustalW* (Thompson *et al.*, 1994 ▸) and amino-acid sequence identity was estimated with *BLAST* (Altschul *et al.*, 1997 ▸) by performing one-to-one pairwise analysis. Primary sequence information was obtained from the Universal Protein Resource (UniProt) (http://www.uniprot.org).

### 2.2. Sample preparation   

The *Hd*_1NES1_NiR gene was ordered from GenScript with the NCBI reference code WP_015596837.1. The N-terminal signal peptide predicted by *Signal-3L* (version 2.0, Zhang & Shen, 2017 ▸) was removed from the ordered gene. The gene with a TEV cleavage site was cloned into pET-26b(+) (Novagen, Darmstadt, Germany) between the NdeI and XhoI sites. The resultant plasmid was verified by DNA sequencing.

An *E. coli* host strain BL21(DE3) cell (New England BioLabs Inc.) was transformed with the plasmid. A single colony was grown in 50 ml Luria–Bertani (LB) medium supplemented with 50 µg ml^−1^ kanamycin and incubated at 37°C for 16 h at 240 rev min^−1^. A 5 ml sample of culture was inoculated into 500 ml of LB medium supplemented with the same concentration of kanamycin and incubated at 37°C at 180 rev min^−1^ until OD_600 nm_ reached ∼0.6. Subsequently, final concentrations of 1.0 m*M* CuSO_4_ and 0.5 m*M* iso­propyl β-d-1-thio­galacto­pyran­oside (IPTG) were added and overexpression was induced at 18°C for 16 h at 180 rev min^−1^. The cells were harvested by centrifugation (4690*g*, 45 min, 4°C). The pellet was washed with 50 ml phosphate-buffered saline (PBS) pH 7.4 and harvested by centrifugation (3140*g*, 30 min, 4°C).

The cells were suspended in 50 ml of lysis buffer 100 m*M* Tris–HCl pH 8.0, 500 m*M* NaCl, 10 m*M* imidazole containing a protease inhibitor tablet (Roche) for 1 l culture. After lysozyme was added to a final concentration of 0.5 mg ml^−1^, the suspension solution was incubated on ice for 20 min. The cells were disrupted by sonication on ice. The cell debris was removed by centrifugation (29 900*g*, 45 min, 4°C). The supernatant was filtered and applied to a 5 ml of His-tag affinity column HisTrap^TM^ HP (GE Healthcare, Buckinghamshire, UK) equilibrated with the lysis buffer. The resin was washed with the same buffer and the protein was eluted with 10 ml of elution buffer 100 m*M* Tris–HCl pH 8.0, 500 m*M* NaCl, 250 m*M* imidazole. The elution solution was dialyzed at 4°C for 24 h against size-exclusion chromatography (SEC) buffer 100 m*M* Tris–HCl pH 8.0, 500 m*M* NaCl, 10%(*v*/*v*) glycerol. After dialysis, a final concentration of 2 m*M* DTT was added and the protein was incubated with TEV protease (50:1) at 4°C for 16 h to remove the 6×His-tag. The protein solution was concentrated and applied on an SEC column HiLoad 16/600 Superdex 200 pg (GE Healthcare, Buckinghamshire, UK) equilibrium with SEC buffer. The protein was eluted at a flow rate of 1.0 ml min^−1^. The elution fractions were dialyzed at 4°C for 16 h against Cu-loading buffer, SEC buffer with 1.0 m*M* CuSO_4_, to reconstitute the T2Cu site. After dialysis, the protein solution was concentrated and applied again on the same SEC column equilibrated with SEC buffer. The protein was eluted at a flow rate of 1.0 ml min^−1^. The elution fractions were concentrated and stored at −80°C. All chromatography steps were performed at 4°C.

### 2.3. UV–visible absorption spectrum measurement   

UV–visible absorption spectra were recorded at room temperature on a Cary 300 Bio UV–visible spectrophotometer (Varian, Palo Alto, USA). *Hd*_1NES1_NiR was prepared at 1.0 mg ml^−1^ in 100 m*M* Tris–HCl pH 8.0, 500 m*M* NaCl, 10%(*v*/*v*) glycerol for spectral measurements.

### 2.4. Oligomeric state analysis   

The molecular mass of *Hd*_1NES1_NiR was estimated by comparison with retention volumes of marker proteins (GE Healthcare, Buckinghamshire, UK). The marker proteins, blue dextran (2000 kDa), thyroglobulin (669 kDa), ferritin (440 kDa), aldolase (158 kDa), conalbumin (75 kDa) and ovalbumin (44 kDa), dissolved in SEC buffer 100 m*M* Tris–HCl pH 8.0, 500 m*M* NaCl, 10%(*v*/*v*) glycerol were applied on an SEC column HiLoad 16/600 Superdex 200 pg (GE Healthcare, Buckinghamshire, UK) equilibrium with SEC buffer and eluted at a flow of 1.0 ml min^−1^. A calibration curve [*K*_av_ value versus log(Mw) where Mw = molecular weight] for these marker proteins was obtained. The *K*_av_ value is defined with the equation *K*_av_ = (*V*_e_ − *V*_o_)/(*V*_c_ − *V*_o_), where *V*_e_, *V*_o_ and *V*_c_ are the elution, column void and geometric column volumes, respectively. The molecular mass was estimated with their *V*_e_ values obtained from the calibration curve.

### 2.5. NiR activity measurement   

NiR activity was assessed under anaerobic conditions using an NO-detectable ISO-NOP electrode (World Precision Instruments, Serasota, USA). The 3 ml of assay mixture containing nitro­gen saturated 50 m*M* HEPES buffer (pH 6.5), 8.0 m*M* sodium ascorbate, 80 µ*M* phenazine metho­sulfate (PMS) and 8.0 m*M* sodium nitrite was prepared in the vessel under anaerobic conditions. The electrode was inserted into the mixture and the baseline voltage was confirmed to be constant for 1 min. The reaction was initiated by the addition of a tiny volume of the *Hd*_1NES1_NiR sample at a final concentration of 300 n*M* and the time-course of NO production [voltage (V) versus time (s)] was monitored. The activity value [nmol s^−1^(nmol of protein)^−1^] for a linear slope was estimated using the experimentally determined calibration curve [voltage (V) versus NO production (nmol)].

### 2.6. Structure determination   

For the as-isolated structure, the *Hd*_1NES1_NiR sample in 20 m*M* Tris–HCl pH 7.5 was concentrated to 20 mg ml^−1^. The protein was crystallized by the hanging-drop vapor-diffusion method: 2 µl of sample solution was mixed with 1 µl of crystallization reagent 20%(*w*/*v*) PEG 1000, 0.1 *M* sodium citrate tribasic dihydrate pH 5.5, 0.1 *M* lithium sulfate monohydrate and equilibrated over 200 µl of the crystallization reagent at room temperature. The crystal was transferred in 20%(*w*/*v*) PEG 1000, 0.1 *M* sodium citrate tribasic dihydrate pH 5.5, 0.1 *M* lithium sulfate monohydrate, 20%(*v*/*v*) ethyl­ene glycol and flash-cooled in liquid nitro­gen.

For the substrate/product-bound structure, the *Hd*_1NES1_NiR sample in 20 m*M* Tris–HCl pH 7.5 was concentrated to 20 mg ml^−1^. The protein was crystallized by the hanging-drop vapor-diffusion method: 1 µl of sample solution was mixed with 1 µl of crystallization reagent 22.5%(*v*/*v*) PEG Smear Low (Chaikuad *et al.*, 2015 ▸), 0.1 *M* sodium cacodylate pH 5.3, 0.2 *M* ammonium nitrate and equilibrated over 200 µl of the crystallization reagent at room temperature. The crystal was transferred in 22.5%(*v*/*v*) PEG Smear Low, 0.1 *M* sodium cacodylate pH 5.3, 0.2 *M* ammonium nitrate, 100 m*M* NaNO_2_, 20%(*v*/*v*) glycerol and flash-cooled in liquid nitro­gen.

Diffraction data were collected at the I04 beamline, Diamond Light Source, UK, at 100 K using an EIGER X 16M detector. The diffraction images were processed with *DIALS* (Winter *et al.*, 2018 ▸) in *XIA2* (Winter, 2010 ▸) and *AIMLESS* (Evans & Murshudov, 2013 ▸) for the as-isolated structure, and with a combination of *autoPROC* (Vonrhein *et al.*, 2011 ▸) and *STARANISO* (Tickle *et al.*, 2018 ▸) for the substrate/product-bound structure, both in space group *P*6_5_22. For the substrate/product-bound crystal, an additional data set was collected at 1.33 Å wavelength to confirm the correct Cu incorporation. For the as-isolated structure, the initial model was obtained by molecular replacement with *MOLREP* (Vagin & Teplyakov, 2010 ▸) using the structure of the trimer *Hd*_A3151_NiR (PDB entry 2dv6). The substrate/product-bound structure was refined directly from the as-isolated structure. The models were refined with *REFMAC5* (Murshudov *et al.*, 2011 ▸) in *CCP4* (Winn *et al.*, 2011 ▸) and manually rebuilt with *Coot* (Emsley *et al.*, 2010 ▸). The quality of the final models was assessed with *MolProbity* (Chen *et al.*, 2010 ▸). Data collection and structure refinement statistics are summarized in Table 1 ▸. Sequence alignment was performed with *TM-align* (Zhang & Skolnick, 2005 ▸). Structural figures were prepared using *PyMOL* (v.1.4; Schrödinger).

## 3. Results   

### 3.1. Spectroscopic and functional characterization of *Hd*_1NES1_NiR   

We have purified the N-terminal cupredoxin-tethered three-domain CuNiR from *Hyphomicrobium denitrificans* strain 1NES1 (*Hd*_1NES1_NiR), which is the same class of enzyme as the structurally characterized CuNiR from *Hyphomicrobium denitrificans* strain A3151 (*Hd*_A3151_NiR) (Nojiri *et al.*, 2007 ▸). The primary structure alignment shows 84% sequence identity between these with complete conservation of the ligand residues to the catalytic core T1Cu and T2Cu centers and the active-site residues Asp_CAT_ and His_CAT_ involved in substrate-anchoring and catalysis (Fig. S1 of the supporting information). The different amino-acid residues are predominantly distributed on the N-terminal tethered cupredoxin domain and the N-terminal and signal peptides. The UV–visible absorption spectrum revealed *Hd*_1NES1_NiR to have an *A*_600_/*A*_460_ ratio of ∼1.6 [Fig. S2(*a*)] compared with ∼1.9 for *Hd*_A3151_NiR (Deligeer *et al.*, 2002 ▸). These spectral features have been assigned to S(Cys)-to-Cu^ll^ charge transfer transitions, the intensity of which depends on the detailed geometry of the T1Cu site. In the case of *Hd*NiRs, the difference in this ratio results in a stronger greenish appearance of *Hd*_1NES1_NiR and is the result of the structural differences in the T1Cu_N_ sites of the two *Hyphomicobium* species. The MW of *Hd*_1NES1_NiR determined by size-exclusion chromatography is consistent with it being a hexamer [Fig. S2(*b*)]. The broad elution peak may indicate the presence of additional unresolved lower oligomeric states. The NiR activity assayed by direct measurements of NO formation demonstrated that the specific NiR activity of *Hd*_1NES1_NiR is 8.3 ± 0.8 nmol s^−1^ (nmol of protein)^−1^ [Fig. S2(*c*)], which is ∼40-fold less than that reported for *Hd*_A3151_NiR (Deligeer *et al.*, 2002 ▸). Unexpectedly, the time course assay profile for NO production by *Hd*_1NES1_NiR exhibited a lag period of ∼200 s before the rate became linear when the reaction was initiated by the addition of the enzyme to the assay mixture, but was eliminated when *Hd*_1NES1_NiR was pre-incubated with substrate (nitrite) [Fig. S2(*d*)] or reductant (ascorbate) [Fig. S2(*e*)]. The specific activity without pre-incubation (1.7 ± 0.2) was increased by approximately fivefold more when pre-incubated with substrate (8.3 ± 0.8), whereas with reductant showed it a marginal increase (2.6 ± 0.3).

### 3.2. Crystallographic structure of as-isolated *Hd*_1NES1_NiR   

**Figure 1:**
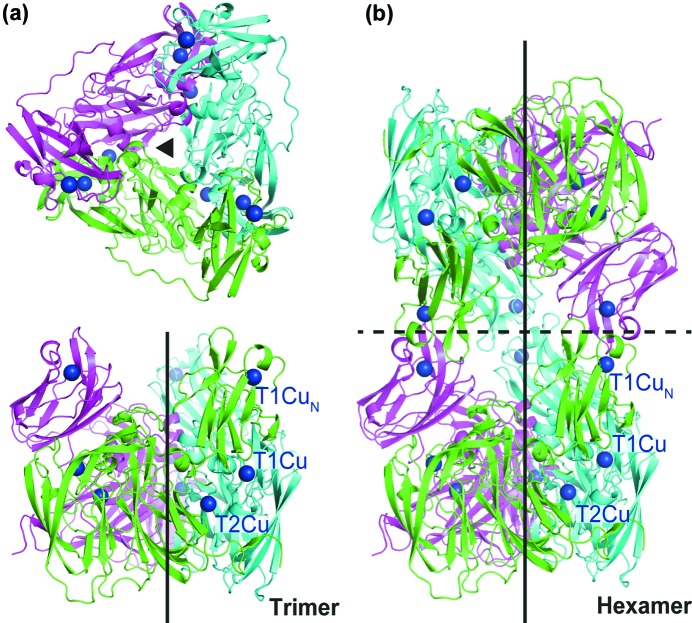
Overall structures of *Hd*_1NES1_NiR in trimeric and hexameric forms. (*a*) Top (upper) and side (lower) views of a trimeric *Hd*_1NES1_NiR, colored green, magenta and cyan for each monomer. Threefold axis symmetry is indicated by a black closed triangle (upper) and black line (lower). The T1Cu and T2Cu ions in the core domain and T1Cu_N_ ion in the extra cupredoxin domain are shown by deep-blue spheres. (*b*) Side view of a hexameric *Hd*_1NES1_NiR coloured green, magenta and cyan for each monomer generated by crystallographic symmetry. Threefold axis symmetry for each trimer is indicated by a black line. The interaction interface between the two trimers through extra cupredoxin domains is indicated by a black broken line. The T1Cu and T2Cu ions in the core domain and T1Cu_N_ ion in the extra cupredoxin domain are represented by deep-blue spheres.

The crystallographic structure of *Hd*_A3151_NiR isolated from the native source was reported more than a decade ago as the first and only structure of an N-terminal tethered three-domain CuNiR (Nojiri *et al.*, 2007 ▸). It remained the only hexameric structure for a CuNiR where the tethered domain T1Cu was placed too far away from the T1Cu–T2Cu NiR core, hence, casting doubt on its role in electron transfer. It has thus remained imperative to discover another representative of this class of NiR and determine its high-resolution structure in order to resolve the details of structure–function relationships in this class of CuNiRs. With this goal in mind we have determined the crystallographic structure of *Hd*NiR from a different strain, 1NES1 (*Hd*_1NES1_NiR), at 2.05 Å resolution using protein heterologously expressed in *E. coli* (Fig. 1 ▸, Table 1 ▸).

The overall structure of a monomer is quite similar to that of *Hd*_A3151_NiR with an r.m.s.d. value of 0.64 (Cαs) for 422 amino-acid residues. The asymmetric unit of the crystal (space group *P*6_5_22) contains a trimer, which creates a hexamer with a second symmetry related trimer [Fig. 1 ▸(*b*)], also observed for *Hd*_A3151_NiR (PDB entry 2dv6; Nojiri *et al.*, 2007 ▸). In both of these *Hd*NiRs, the T1Cu_N_ in the N-terminal tethered cupredoxin domain is placed at a distance of ∼24 Å from the catalytic core T1Cu, which may be considered too far away for an effective electron transfer. Nevertheless, pulse radiolysis data for *Hd*_A3151_NiR, which is also hexameric in solution, has shown that in the presence of nitrite, type-1 Cu_N_ receives the electron first and then passes it onto the type-1 Cu_core_ of the adjacent monomer ready for catalysis (Nojiri *et al.*, 2007 ▸).

A close examination of the structural differences between the two *Hd*NiRs was made with particular emphasis on the non-conserved amino-acid residues between the two enzymes. These residues are predominantly located on the surface of the N-terminal tethered cupredoxin domain, which is exposed to solvent [Fig. S3(*c*)], though some are also in the inter- and/or intra monomer interface (Fig. S4). The substitutions in this domain in *Hd*_1NES1_NiR result in a much less negative and water-inaccessible surface compared with *Hd*_A3151_NiR [Figs. S3(*a*) and S3(*b*)]. Differences are also observed in the water networks near T1Cu_core_ and T1Cu_N_ in the catalytic core and N-terminal tethered cupredoxin domains [Figs. S3(*d*) and S4(*d*)], respectively, together with a number of non-conserved amino-acid residues between the two *Hd*NiRs. In both T1Cu sites in *Hd*_1NES1_NiR, a bridging water molecule expected to be hydrogen-bonded to the ligand histidine His271 and His122 (for T1Cu_core_ and T1Cu_N_, respectively) is missing [Figs. S3(*d*) and S4(*d*)], which may also contribute to the UV-spectral difference between the two *Hd*NiRs together with subtle changes in the T1Cu_N_ geometry (Deligeer *et al.*, 2002 ▸).

## 4. Structure of the tethering linker, N-terminal peptide and catalytic T2Cu site of *Hd*_1NES1_NiR   

**Figure 2:**
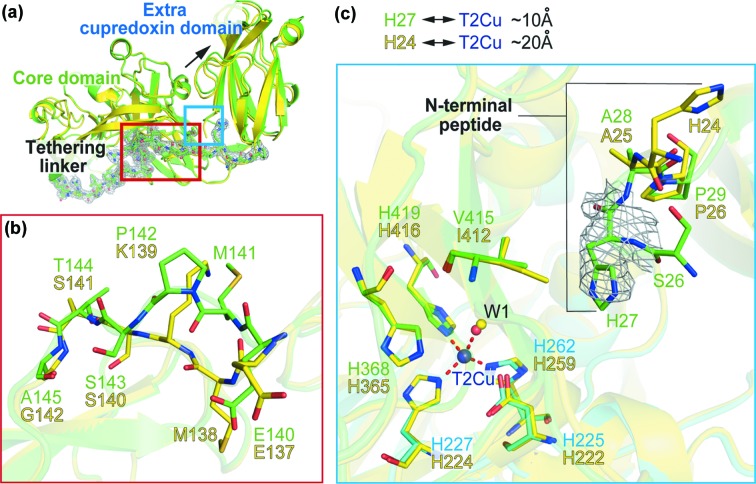
Structural differences in the tethering linker and N-terminal peptide between *Hd*_1NES1_NiR and *Hd*_A3151_NiR. (*a*) Monomer of *Hd*_1NES1_NiR colored green superimposed on the core domain of *Hd*_A3151_NiR colored yellow. The monomer is constructed with the core domain, extra cupredoxin domain and tethering linker between them. The 2*F*_o_ *F*_c_ electron-density map at the 1.0σ level is shown for the tethering linker. The main-chain structural difference between the two *Hd*NiRs is indicated by a black arrow. (*b*) The middle part of the tethering linker and (*c*) the N-terminal peptide near the T2Cu of *Hd*_1NES1_NiR colored green superimposed on the core domain of *Hd*_A3151_NiR coloured yellow. The T2Cu ion in the core domain is represented by a deep-blue sphere. The ligand water (W1) molecules for *Hd*_1NES1_NiR and *Hd*_A3151_NiR are represented by red and yellow spheres, respectively. Coordination to the T2Cu ion is shown by a red broken line. The 2*F*_o_ *F*_c_ electron density map at the 1.0σ level is shown for the His27 of *Hd*_1NES1_NiR. The distances between His27 and His24 and T2Cu are indicated (∼10 and ∼20 Å for 1NES1 and A3151, respectively).

Structural differences are also observed in the central part of the tethering linker between the catalytic core domain and N-terminal tethered cupredoxin domain in the two *Hd*NiRs (Fig. 2 ▸). Lys139 and Gly142 of *Hd*_A3151_NiR are replaced by Pro142 and Ala145 in *Hd*_1NES1_NiR, increasing the rigidity of the main chain. This results in a different orientation of the side chains of Glu140 and Met141 in *Hd*_1NES1_NiR [Fig. 2 ▸(*b*)]. These residues are located in the middle part of the tethering linker, which is between the outside flexible loop exposed to solvent and the inner region that interacts with the core domain [Fig. 2 ▸(*a*)]. The N-terminal tethered cupredoxin domain of *Hd*_1NES1_NiR is positioned ∼1.0 Å away from the core domain compared with *Hd*_A3151_NiR, probably arising from combinatorial structural differences in this region.

Despite the fact that *Hd*NiR from two different strains of bacteria crystallize in different space groups, neither structure shows electron density for the N-terminal peptide, reflecting the intrinsic high flexibility of the region. The two structures show visible electron density starting from an equivalent residue at the N-terminus, His27 and His24 for *Hd*_1NES1_NiR and *Hd*_A3151_NiR, respectively. However, they differ significantly in conformation consistent with the flexibility of the region [Fig. 2 ▸(*c*)]. The His27 of *Hd*_1NES1_NiR is positioned closer to the T2Cu at a distance of ∼10 Å, compared with ∼20 Å in *Hd*_A3151_NiR. This difference likely represents a different conformational state of the tethering linker [Fig. 2 ▸(*a*)] of the *Hd*NiR enzymes. The different position of the histidine could be derived from the substitution of Ile412 in *Hd*_A3151_NiR by the less bulky valine in *Hd*_1NES1_NiR. Like the C-terminal tethered *Rp*NiR, the dynamic features of the linker may be important for communication between the redox center of the tethered domain and the core NiR (Hedison *et al.*, 2019 ▸).

## 5. Structures of substrate- and product-bound forms of *Hd*_1NES1_NiR trapped in the same nitrite-soaked crystal   

**Figure 3:**
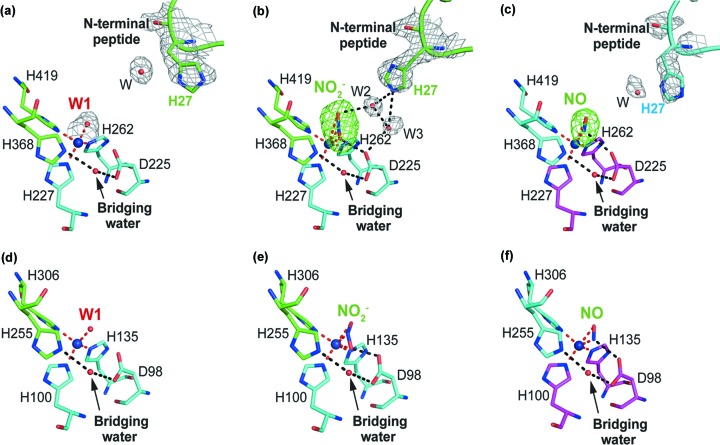
Ligand-bound structures of *Hd*_1NES1_NiR compared with those of *Ac*NiR. (*a*) Ligand water (W1)-, (*b*) nitrite (NO_2_^−^)- and (*c*) nitric oxide (NO)-bound T2Cu of *Hd*_1NES1_NiR colored green, magenta and cyan for each monomer. The T2Cu ion is represented by a deep-blue sphere. The water molecules are represented by red spheres and the bridging water is indicated by a black arrow. Coordination to the T2Cu ion is shown by a red broken line and the interaction is shown by a black broken line. The *F*_o_ *F*_c_ electron density map at the 5.0σ level is shown for nitrite (NO_2_^−^) and nitric oxide (NO). The 2*F*_o_ *F*_c_ electron-density map at the 1.0σ level is shown for the His27, the ligand-water (W1) and the other waters (W2, W3, W). (*d*) The ligand water (W1)-, (*e*) nitrite (NO_2_^−^)- and (*f*) nitric oxide (NO)-bound T2Cu of the *Ac*NiR are colored green and cyan for each monomer. The T2Cu ion is represented by a deep-blue sphere. The water molecules are represented by red spheres and the bridging water is indicated by a black arrow. Coordination to the T2Cu ion is shown by a red broken line and the interaction is shown by a black broken line. The structural coordinates for (*d*), (*e*) and (*f*) are from the PDB entries 6gsq and 6gto (Halsted *et al.*, 2019 ▸), and 5of8 (Horrell *et al.*, 2018 ▸), respectively.

None of the C- or N-terminal cytochrome or cupredoxin tethered CuNiRs have demonstrated successful soaking of substrate into the crystals. Using simple soaking of *Hd*_1NES1_NiR crystals, the substrate-bound structure has been determined to 2.1 Å resolution. Intriguingly, three T2Cu sites belonging to the trimer in the asymmetric unit are occupied by different ligands: two by the substrate nitrite and one by a diatomic molecule consistent with it being the product nitric oxide. Each of these independent sites have full occupancy of copper and the ligand (Fig. 3 ▸).

Thus, we are able to compare for the first time a tethered CuNiR with two-domain NiRs in three different catalytically important states, namely the as-isolated water (W1)-bound structure and the substrate (NO_2_^−^)- and product (NO)-bound structures. Interestingly, comparison among these shows different structural arrangements around the T2Cu including the positioning of His27 in the flexible N-terminal peptide. For the ligand water (W1)-bound structure [Fig. 3 ▸(*a*)], the N-terminal His27 assumes an outward open conformation, where a single water molecule is positioned near the His27. The relative position of the W1 to the T2Cu with a W1–T2Cu–His419 angle of ∼90° is very similar to that observed in equivalent structures from other classical two-domain CuNiRs such as *Ac*NiR [Fig. 3 ▸(*d*)], suggesting that the displacement by substrate should be similarly favorable. The NO_2_^−^-bound structure [Figs. 3 ▸(*b*) and S5(*a*)] shows a remarkable inward pointing of His27 towards the T2Cu and its closed conformation involving W2–W3 mediation. This suggests it plays a role in anchoring the substrate. The nitro­gen atom of His27 is linked to bound NO_2_^−^, mediated by the second water (W2), as well as to Asp225 (Asp_CAT_), mediated by the third water (W3). The NO_2_^−^ is bound to the T2Cu in a side-on conformation via a single nitro­gen atom and a single proximal oxygen atom with distances of 1.9 and 2.0 Å, respectively. The distal oxygen atom of the NO_2_^−^ with a longer distance of 3.0 Å to the T2Cu is linked to the nitro­gen atom of His27. The Asp_CAT_ forms a hydrogen bond to the proximal oxygen atom of the NO_2_^−^ with a distance of 2.5 Å. The His368 (His_CAT_) residue also forms a hydrogen bond to the bridging water [Figs. 3 ▸(*b*) and S5(*a*)], which is located at the terminus of the proton channel extending from the solvent region (see below). This is not the case in the water (W1)- and NO-bound structures [Figs. 3 ▸(*a*), 3 ▸(*c*) and S5(*b*)]. In the case of the NO_2_^−^-bound structure, W2 and W3 form a hydrogen bond to each other with a distance of 2.7 Å [Figs. 3 ▸(*b*) and S5(*a*)].

For the NO-bound structure [Figs. 3 ▸(*c*) and S5(*b*)], again, the N-terminal His27 assumes an outward opened conformation directed towards the solvent and consequently loses water-mediated linkages to the ligand and Asp_CAT_. In this case both the water and His27 itself show poorer electron density compared with the other two structures, suggesting higher flexibility of the N-terminal peptide that may facilitate release of the NO from the T2Cu with consequent return to the resting state. The corresponding histidine in *Hd*_A3151_NiR (His24 of *Hd*_A3151_NiR) has a more open conformation placing it quite far from the T2Cu [Fig. 2 ▸(*c*)]. The NO is bound to the T2Cu in a side-on manner similar to that observed for two-domain *Ac*NiR [Fig. 3 ▸(*f*)] and with N and O at 2.0 and 2.5 Å with a tilt angle of 30°. The nitro­gen atom is located 3.1 Å from the closest side-chain oxygen atom of Asp225. Unlike the NO_2_^−^-bound structure [Figs. 3 ▸(*b*) and S5(*a*)], Asp225 forms a hydrogen bond with water in the proton channel with a distance of 2.8 Å [Figs. 3 ▸(*c*) and S5(*b*)]. We note that the unprecedented crystallographic observations of side-on NO binding geometry in CuNiR (Antonyuk *et al.*, 2005 ▸; Tocheva *et al.*, 2004 ▸) have been treated with scepticism by the chemical biology and synthetic chemistry communities until very recently (Ghosh *et al.*, 2007 ▸; Bower *et al.*, 2019 ▸). The observation of side-on NO binding geometry seen here adds to the gathering evidence that this geometry is energetically stable at the T2Cu site of CuNiR and is an intrinsic part of the catalytic turnover.

## 6. Structure of the proton channel and the alternative electron transfer pathway of *Hd*_1NES1_NiR   

**Figure 4:**
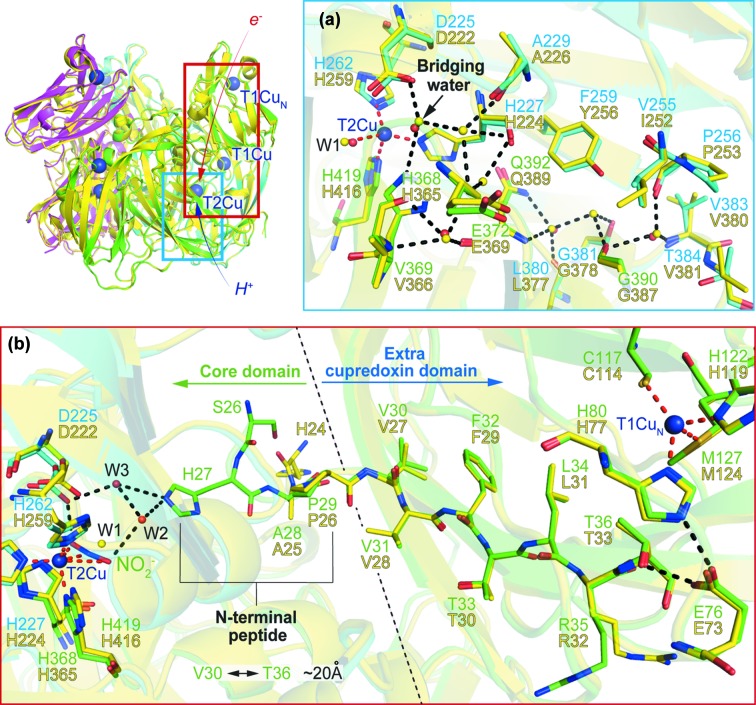
Structural differences in the proton channel and structural preservation of the alternative electron transfer route between *Hd*_1NES1_NiR and *Hd*_A3151_NiR. (*a*) Proton channel of *Hd*_1NES1_NiR colored green and cyan for each monomer superimposed on *Hd*_A3151_NiR colored yellow for all monomers for simplicity. The T2Cu ion in the core domain is represented by a deep-blue sphere. The water molecules for *Hd*_1NES1_NiR and *Hd*_A3151_NiR are represented by red and yellow spheres, respectively, and the bridging water is indicated by a black arrow. Coordination to the T2Cu ion is shown by a red broken line and the interaction is shown by a black broken line. (*b*) Alternative electron transfer route of *Hd*_1NES1_NiR colored green and cyan for each monomer superimposed on the extra cupredoxin domain of *Hd*_A3151_NiR colored yellow for all monomers for simplicity. The T1Cu_N_ ion in the extra cupredoxin domain and T2Cu ion in the core domain are represented by deep-blue spheres. The mediation water molecules (W2 and W3) and ligand water molecule (W1) for *Hd*_1NES1_NiR and *Hd*_A3151_NiR are shown by red and yellow spheres, respectively. Coordination to the T1Cu_N_ and T2Cu ions is shown by a red broken line and the interaction is shown by a black broken line. The distance between Val30 and Thr36 is indicated (∼20 Å).

The proton channel, which extends from the solvent to the catalytic T2Cu at the inter-monomer interface, is different in the two *Hd*NiRs [Fig. 4 ▸(*a*)]. Val381 of *Hd*_A3151_NiR, which is located at the entrance to the channel, is replaced with the more hydro­philic threonine (Thr384), whilst Tyr256 is replaced with the more hydro­phobic phenyl­alanine (Phe259 of *Hd*_1NES1_NiR). Ile252 of *Hd*_A3151_NiR is replaced with the less bulky valine (Val255 of *Hd*_1NES1_NiR). More importantly, the three water molecules which form a proton pathway in *Hd*_A3151_NiR are missing in *Hd*_1NES1_NiR. These structural differences may contribute to the lower NiR activity of *Hd*_1NES1_NiR.

We have investigated an alternative ET route from the T1Cu_N_ in the tethered cupredoxin domain to the T2Cu in the core domain *via* long-range electron tunneling through a β-strand polypeptide over a distance of ∼16 to 26 Å, as has been reported for Ru-labeled cupredoxin by Gray & Winkler (2005 ▸). The completely conserved hydro­phobic β-strand is present in the structures of the two *Hd*NiRs, starting from Val30 to Thr36 (for *Hd*_1NES1_NiR) with a distance of ∼20 Å within this region, suggesting the possibility for electron transfer through this strand. The structurally conserved glutamic acid (Glu76 and Glu73 of *Hd*_1NES1_NiR and *Hd*_A3151_NiR, respectively) forms a hydrogen bond with threonine (Thr36 and Thr33) with a distance of ∼2.7 Å as well as with the ligand histidine to the T1Cu_N_ (His80 and His77) with a distance of ∼2.6 Å. We suggest a role for this residue in making an electron-transfer bridge between the T2Cu_N_ and Thr36, the starting residue of the probable electron tunneling hydro­phobic β-strand. Val30, the terminal residue of this β-strand, connects to the T2Cu through the N-terminal peptide including the NO_2_^−^-capturing His27 and the W2–W3 water mediation system in the NO_2_^−^-bound structure of *Hd*_1NES1_NiR. This suggests that electron transfer from the T1Cu_N_ in the tethered cupredoxin domain to the T2Cu in the core domain is dependent on both the presence of substrate and the dynamics of His27 movement. Pulse radiolysis data for *Hd*_A3151_NiR indicate that ET only occurs in the presence of NO_2_^−^and that the electron preferentially goes to the T1Cu_N_. The mutational studies of the T1Cu_core_ of *Hd*_A3151_NiR revealed that the T1Cu-deficient enzyme still exhibited a significant NiR activity (Yamaguchi *et al.*, 2004 ▸). This can now be rationalized neatly by the proposed long-range ET through the hydro­phobic β-strand to the catalytic T2Cu site.

## 7. Discussion   

Among nearly a couple of hundred structures of CuNiRs in the Protein Data Bank (PDB) we provide the second example of an enzyme with a hexameric rather than trimeric structure. The hexameric structure observed for the N-terminal cupredoxin-tethered three-domain CuNiR from two strains of *Hyphomicrobium denitrificans* suggests that it is likely to offer an advantage for these organisms. In both of the hexameric structures of *Hd*_1NES1_NiR and *Hd*_A3151_NiR, the T1Cu_N_ in the N-terminal tethered cupredoxin domain is placed too far away from the catalytic core T1Cu for effective electron transfer (Fig. 1 ▸) (Nojiri *et al.*, 2007 ▸), suggesting that conformational changes may be required to place the tethered cupredoxin domain close to the core domain or an alternative mechanism is at work. Differences in the proton channel between the two *Hd*NiRs are also likely to contribute to these differences.

Though several C-terminal and N-terminal tethered CuNiRs have been identified and structurally characterized, the scarcity of substrate/product-bound structures has hampered understanding of the role of tethered partner electron donor proteins. When these enzymes were discovered it was simply assumed that tethering would provide functional advantage by narrowing the range of conformational searches that are generally required in encounter complexes. We have successfully determined the substrate-bound structure *Hd*_1NES1_NiR, obtained by simple soaking of an as-isolated enzyme crystal. This represents the first substrate-bound structure of the wild-type tethered CuNiR.

The nitrite-soaked crystal trapped the substrate in two of the monomers while the third monomer showed the product (NO) with full occupancy. The product may have formed from enzyme turnover catalyzed by solvated electrons generated during X-ray data collection, but the full occupancy of a single species is surprising. This suggests a very slow off-rate for dissociation of the product which is consistent with the lower NiR activity in these enzymes. Our data provides clear evidence for the role of the N-terminal peptide that carries His27 (Fig. 3 ▸) in water-mediated anchoring of the substrate at the T2Cu site. The conformational flexibility of this N-terminal peptide and His27 may be critical for the substrate entry, anchoring and product formation stages of the catalytic reaction, requiring it to move to the outward conformation for the eventual release of the product. Our data also provide an explanation for the significant activity for the core T1Cu mutant for *Hd*_A3151_NiR by identifying a long-range electron tunneling route via a hydro­phobic β-strand, thereby bypassing the T1Cu_core_ and delivering electrons directly to the catalytic T2Cu center.

## 8. Data availability   

The atomic coordinates and structure factors of *Hd*_1NES1_NiR have been deposited in the Protein Data Bank (http://www.rcsb.org/) under the accession codes 6tfo and 6tfd for the as-isolated and substrate/product-bound structures, respectively.

## Supplementary Material

Supporting information file. DOI: 10.1107/S2052252520005230/jt5044sup1.pdf

PDB reference: *Hd*_1NES1_NiR (as-isolated), 6tfo

PDB reference: *Hd*_1NES1_NiR (nitrite-soaked), 6tfd

## Figures and Tables

**Table 1:** Data collection and refinement statistics Numbers in parentheses represent the value for the lowest/highest resolution shell (innermost/outermost shells).

	*Hd*_1NES1_NiR (as-isolated)	*Hd*_1NES1_NiR (nitrite-soaked)
Ligands	W1	NO_2_/NO
Data collection		
Space group	*P*6_5_22	*P*6_5_22
Wavelength (Å)	0.9795	0.9795
*a*, *b*, *c* (Å)	77.07, 77.07, 754.55	77.72, 77.72, 758.20
Resolution (Å)	66.75–2.05 (66.75–10.85/2.09–2.05)	126.37–2.10 (126.37–5.7/2.14–2.10)
No. of reflections, total/unique	363227/85026	987567/81889
*R*_merge_ † (%)	12.1 (2.9/92.6)	21.6 (6.2/284.5)
*R*_p.i.m._ ‡ (%)	8.4 (1.9/67.7)	6.4 (2.0/82.8)
*I/*σ(*I*)	3.5 (9.5/0.8)	7.7 (24.0/0.9)
CC_1/2_	0.995 (0.998/0.610)	0.998 (0.998/0.426)
Completeness (%)	98.7 (99.8/98.2)	100 (100/100)
Multiplicity	4.3 (3.4/4.4)	12.1 (10.6/12.3)
Refinement		
*R*_work_ §/*R*_free_ ¶	0.227/0.279	0.172/0.227
Resolution (Å)	66.75–2.05	126.37–2.25
No. of atoms		
Protein	9630	9625
Ligand/ion	9	17
Water	528	639
Average *B* factor (Å^2^)		
Protein	51.8	54.9
Ligand/ion	47.1	52.3
Water	50.5	52.6
R.m.s. deviations		
ond lengths (Å)	0.007	0.007
Bond angles (°)	1.511	1.601
Ramachandran plot		
Favored (%)	96.6	97.2
Allowed (%)	99.8	99.7
PDB entry	6tfo	6tfd

†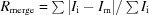, where *I*_i_ is the intensity of the measured reflection and *I*_m_ is the mean intensity of all symmetry related reflections.

‡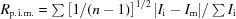 where *I*_i_ is the intensity of the measured reflection, *I*_m_ is the mean intensity of all symmetry related reflections and *n* is the redundancy.

§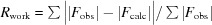, where *F*_obs_ and *F*_calc_ are the observed and calculated structure factors, respectively.

¶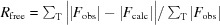, where T is a test data set of 5% of the total reflections randomly chosen and set aside prior to refinement.

## References

[bb1] Altschul, S. F., Madden, T. L., Schäffer, A. A., Zhang, J., Zhang, Z., Miller, W. & Lipman, D. J. (1997). *Nucleic Acids Res.* **25**, 3389–3402. 10.1093/nar/25.17.3389 PMC146917 9254694

[bb2] Antonyuk, S. V., Han, C., Eady, R. R. & Hasnain, S. S. (2013). *Nature*, **496**, 123–126. 10.1038/nature11996 PMC3672994 23535590

[bb3] Antonyuk, S. V., Strange, R. W., Sawers, G., Eady, R. R. & Hasnain, S. S. (2005). *Proc. Natl Acad. Sci.* **102**, 12041–12046. 10.1073/pnas.0504207102 PMC1189323 16093314

[bb4] Boulanger, M. J., Kukimoto, M., Nishiyama, M., Horinouchi, S. & Murphy, M. E. (2000). *J. Biol. Chem.* **275**, 23957–23964. 10.1074/jbc.M001859200 10811642

[bb5] Bower, J. K., Sokolov, A. Y. & Zhang, S. (2019). *Angew. Chem. Int. Ed.* **58**, 10225–10229. 10.1002/anie.201904732 31066187

[bb6] Chaikuad, A., Knapp, S. & von Delft, F. (2015). *Acta Cryst.* D**71**, 1627–1639. 10.1107/S1399004715007968 PMC4528798 26249344

[bb7] Chen, V. B., Arendall, W. B., Headd, J. J., Keedy, D. A., Immormino, R. M., Kapral, G. J., Murray, L. W., Richardson, J. S. & Richardson, D. C. (2010). *Acta Cryst.* D**66**, 12–21. 10.1107/S0907444909042073 PMC2803126 20057044

[bb8] Deligeer, Fukunaga, R., Kataoka, K., Yamaguchi, K., Kobayashi, K., Tagawa, S. & Suzuki, S. (2002). *J. Inorg. Biochem.* **91**, 132–138. 10.1016/s0162-0134(02)00442-7 12121770

[bb9] Dodd, F. E., Van Beeumen, J., Eady, R. R. & Hasnain, S. S. (1998). *J. Mol. Biol.* **282**, 369–382. 10.1006/jmbi.1998.2007 9735294

[bb10] Dong, J., Sasaki, D., Eady, R. R., Antonyuk, S. V. & Hasnain, S. S. (2018). *IUCrJ*, **5**, 510–518. 10.1107/S2052252518008242 PMC6038957 30002851

[bb11] Emsley, P., Lohkamp, B., Scott, W. G. & Cowtan, K. (2010). *Acta Cryst.* D**66**, 486–501. 10.1107/S0907444910007493 PMC2852313 20383002

[bb12] Evans, P. R. & Murshudov, G. N. (2013). *Acta Cryst.* D**69**, 1204–1214. 10.1107/S0907444913000061 PMC3689523 23793146

[bb13] Fukuda, Y., Tse, K. M., Nakane, T., Nakatsu, T., Suzuki, M., Sugahara, M., Inoue, S., Masuda, T., Yumoto, F., Matsugaki, N., Nango, E., Tono, K., Joti, Y., Kameshima, T., Song, C., Hatsui, T., Yabashi, M., Nureki, O., Murphy, M. E., Inoue, T., Iwata, S. & Mizohata, E. (2016). *Proc. Natl Acad. Sci. USA*, **113**, 2928–2933. 10.1073/pnas.1517770113 PMC4801246 26929369

[bb14] Ghosh, S., Dey, A., Usov, O. M., Sun, Y., Grigoryants, V. M., Scholes, C. P. & Solomon, E. I. (2007). *J. Am. Chem. Soc.* **129**, 10310–10311. 10.1021/ja072841c PMC2532526 17685522

[bb15] Godden, J. W., Turley, S., Teller, D. C., Adman, E. T., Liu, M. Y., Payne, W. J. & LeGall, J. (1991). *Science*, **253**, 438–442. 10.1126/science.1862344 1862344

[bb16] Gray, H. B. & Winkler, J. R. (2005). *Proc. Natl Acad. Sci. USA*, **102**, 3534–3539. 10.1073/pnas.0408029102 PMC553296 15738403

[bb17] Halsted, T. P., Yamashita, K., Gopalasingam, C. C., Shenoy, R. T., Hirata, K., Ago, H., Ueno, G., Blakeley, M. P., Eady, R. R., Antonyuk, S. V., Yamamoto, M. & Hasnain, S. S. (2019). *IUCrJ*, **6**, 761–772. 10.1107/S2052252519008285 PMC6608623 31316819

[bb18] Hedison, T. M., Shenoy, R. T., Iorgu, A. I., Heyes, D. J., Fisher, K., Wright, G. S. A., Hay, S., Eady, R. R., Antonyuk, S. V., Hasnain, S. S. & Scrutton, N. S. (2019). *ACS Catal.* **9**, 6087–6099. 10.1021/acscatal.9b01266 PMC7007197 32051772

[bb50] Horrell, S., Kekilli, D., Sen, K., Owen, R. L., Dworkowski, F. S. N., Antonyuk, S. V., Keal, T. W., Yong, C. W., Eady, R. R., Hasnain, S. S., Strange, R. W. & Hough, M. A. (2018). *IUCrJ* **5**, 283–292. 10.1107/S205225251800386X PMC5929374 29755744

[bb19] Kataoka, K., Furusawa, H., Takagi, K., Yamaguchi, K. & Suzuki, S. (2000). *J. Biochem.* **127**, 345–350. 10.1093/oxfordjournals.jbchem.a022613 10731703

[bb20] Murshudov, G. N., Skubák, P., Lebedev, A. A., Pannu, N. S., Steiner, R. A., Nicholls, R. A., Winn, M. D., Long, F. & Vagin, A. A. (2011). *Acta Cryst.* D**67**, 355–367. 10.1107/S0907444911001314 PMC3069751 21460454

[bb21] Nojiri, M. (2016). *Metalloenzymes in Denitrification: Applications and Environmental Impacts*, edited by I. Moura, J. J. G. Moura, S. R. Pauleta & L. B. Maia, pp. 91–113. Cambridge: The Royal Society of Chemistry.

[bb22] Nojiri, M., Koteishi, H., Nakagami, T., Kobayashi, K., Inoue, T., Yamaguchi, K. & Suzuki, S. (2009). *Nature*, **462**, 117–120. 10.1038/nature08507 19890332

[bb23] Nojiri, M., Xie, Y., Inoue, T., Yamamoto, T., Matsumura, H., Kataoka, K., Deligeer, Yamaguchi, K., Kai, Y. & Suzuki, S. (2007). *Proc. Natl Acad. Sci. USA*, **104**, 4315–4320. 10.1073/pnas.0609195104 PMC1838599 17360521

[bb24] Opperman, D. J., Murgida, D. H., Dalosto, S. D., Brondino, C. D. & Ferroni, F. M. (2019). *IUCrJ*, **6**, 248–258. 10.1107/S2052252519000241 PMC6400189 30867922

[bb25] Thompson, J. D., Higgins, D. G. & Gibson, T. J. (1994). *Nucleic Acids Res.* **22**, 4673–4680. 10.1093/nar/22.22.4673 PMC308517 7984417

[bb26] Tickle, I. J., Flensburg, C., Keller, P., Paciorek, W., Sharff, A., Vonrhein, C. & Bricogne, G. (2018). *STARANISO.* Global Phasing Ltd, Cambridge, UK.

[bb27] Tocheva, E. I., Rosell, F. I., Mauk, A. G. & Murphy, M. E. (2004). *Science*, **304**, 867–870. 10.1126/science.1095109 15131305

[bb28] Tsuda, A., Ishikawa, R., Koteishi, H., Tange, K., Fukuda, Y., Kobayashi, K., Inoue, T. & Nojiri, M. (2013). *J. Biochem.* **154**, 51–60. 10.1093/jb/mvt023 23543476

[bb29] Vagin, A. & Teplyakov, A. (2010). *Acta Cryst.* D**66**, 22–25. 10.1107/S0907444909042589 20057045

[bb30] Vonrhein, C., Flensburg, C., Keller, P., Sharff, A., Smart, O., Paciorek, W., Womack, T. & Bricogne, G. (2011). *Acta Cryst.* D**67**, 293–302. 10.1107/S0907444911007773 PMC3069744 21460447

[bb31] Winn, M. D., Ballard, C. C., Cowtan, K. D., Dodson, E. J., Emsley, P., Evans, P. R., Keegan, R. M., Krissinel, E. B., Leslie, A. G. W., McCoy, A., McNicholas, S. J., Murshudov, G. N., Pannu, N. S., Potterton, E. A., Powell, H. R., Read, R. J., Vagin, A. & Wilson, K. S. (2011). *Acta Cryst.* D**67**, 235–242. 10.1107/S0907444910045749 PMC3069738 21460441

[bb32] Winter, G. (2010). *J. Appl. Cryst.* **43**, 186–190.

[bb33] Winter, G., Waterman, D. G., Parkhurst, J. M., Brewster, A. S., Gildea, R. J., Gerstel, M., Fuentes-Montero, L., Vollmar, M., Michels-Clark, T., Young, I. D., Sauter, N. K. & Evans, G. (2018). *Acta Cryst.* D**74**, 85–97. 10.1107/S2059798317017235 PMC5947772 29533234

[bb34] Yamaguchi, K., Kataoka, K., Kobayashi, M., Itoh, K., Fukui, A. & Suzuki, S. (2004). *Biochemistry*, **43**, 14180–14188. 10.1021/bi0492657 15518568

[bb35] Zhang, Y. & Skolnick, J. (2005). *Nucleic Acids Res.* **33**, 2302–2309. 10.1093/nar/gki524 PMC1084323 15849316

[bb36] Zhang, Y. Z. & Shen, H. B. (2017). *J. Chem. Inf. Model.* **57**, 988–999. 10.1021/acs.jcim.6b00484 28298081

[bb37] Zumft, W. G. (1997). *Microbiol. Mol. Biol. Rev.* **61**, 533–616. 10.1128/mmbr.61.4.533-616.1997 PMC232623 9409151

