# Supplementary material for: Structures of substrate- and product-bound forms of a multi-domain copper nitrite reductase shed light on the role of domain tethering in protein complexes
Source: IUCrJ. 2020 Apr 25;7(Pt 3):557–65. doi: 10.1107/S2052252520005230 (PMC7201279; doi:10.1107/S2052252520005230)
Supplement: Supplementary file 1 [file m-07-00557-sup1.pdf]

IUCrJ

Volume 7 (2020)

Supporting information for article:

Structures of substrate and product bound forms of a multi-domain copper nitrite reductase shed light on the role of domain tethering in protein complexes

Daisuke Sasaki, Tatiana F. Watanabe, Robert R. Eady, Richard C. Garratt, Svetlana V. Antonyuk and S. Samar Hasnain

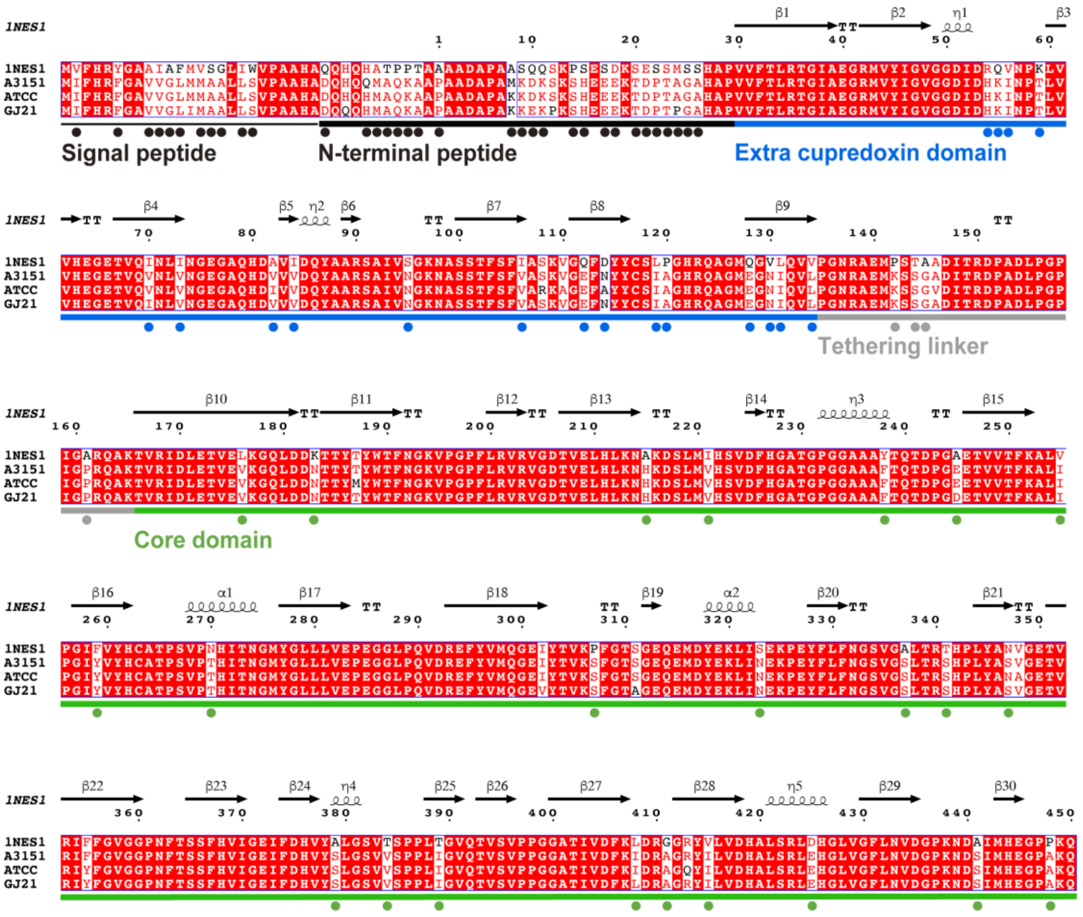

Figure S1 Multiple amino-acid sequence alignment among the N-terminal cupredoxin-tethered three-domain CuNiRs. The N-terminal cupredoxin-tethered three-domain CuNiRs from *Hyphomicrobium denitrificans* strain1NES1(1NES1; UniProt: N0B9M5), *Hyphomicrobium denitrificans* strainA3151(A3151; UniProt: Q8KKH4), *Hyphomicrobium denitrificans* strainATCC 51888(ATCC; UniProt: D8JSS7) and *Hyphomicrobium* sp. strainGJ21(GJ21; UniProt: A0A218PRU1) are aligned. The extra cupredoxin domain and the core domain, which has two cupredoxin domains, and tethering linker between these domains are shown in blue, green and gray lines, respectively. Numbering is consistent with Hd_{1NES1}NiR structure. Secondary structure of Hd_{1NES1}NiRis shown above numbering. The difference amino-acid residues between Hd_{1NES1}NiR and Hd_{A3151}NiR are shown by coloured closed circle. The N-

terminal peptide and signal peptide are indicated. Identical amino-acids are highlighted by white letters in red boxes and similar ones are shown by red letter in white boxes.

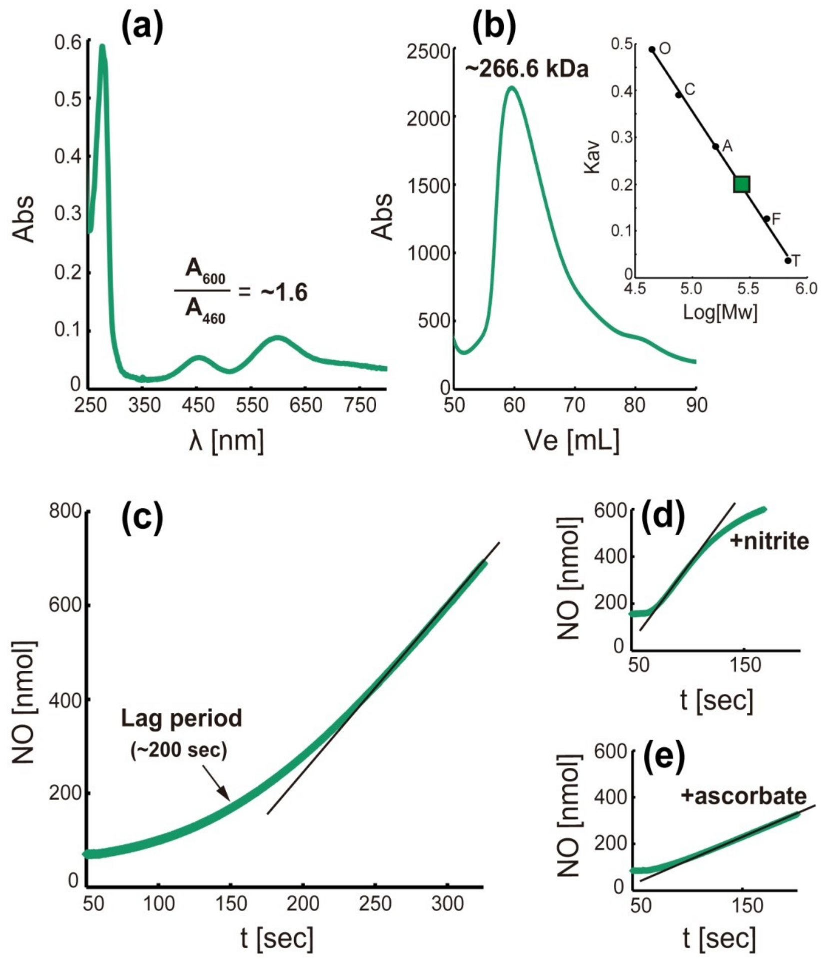

Figure S2 **Functional properties of CuNiR from *Hyphomicrobium denitrificans* strain 1NES1 (*Hd*$_{1NES1}$NiR). (a)** UV-visible absorption profile of the *Hd*$_{1NES1}$NiR. **(b)** Size exclusion chromatography (SEC) profile of the *Hd*$_{1NES1}$NiR. Estimated molecular mass (kDa) of ~266.6 kDa is indicated (Theoretical molecular mass of a monomer is ~49 kDa). Calibration curve with the following standard marker proteins is shown; T: thyroglobulin (669 kDa), F: ferritin (440 kDa), A: aldolase (158 kDa), C: conalbumin (75 kDa), O: ovalbumin (44 kDa). **(c-e)** Time-course NO production (NiR activity) measurement profiles of the *Hd*$_{1NES1}$NiR. The enzyme pre-

incubated with nitrite or ascorbate for 600 sec before injection are shown in (d) and (e), respectively. The lag period (~200 sec) without pre-incubation is indicated in (c). Protein sample, ascorbate, and nitrite injection positions are at 60 sec. Specific NiR activity values [nmol sec^{-1} (nmol of protein)$^{-1}$] estimated with linear slope (black lines) are describe in main text.

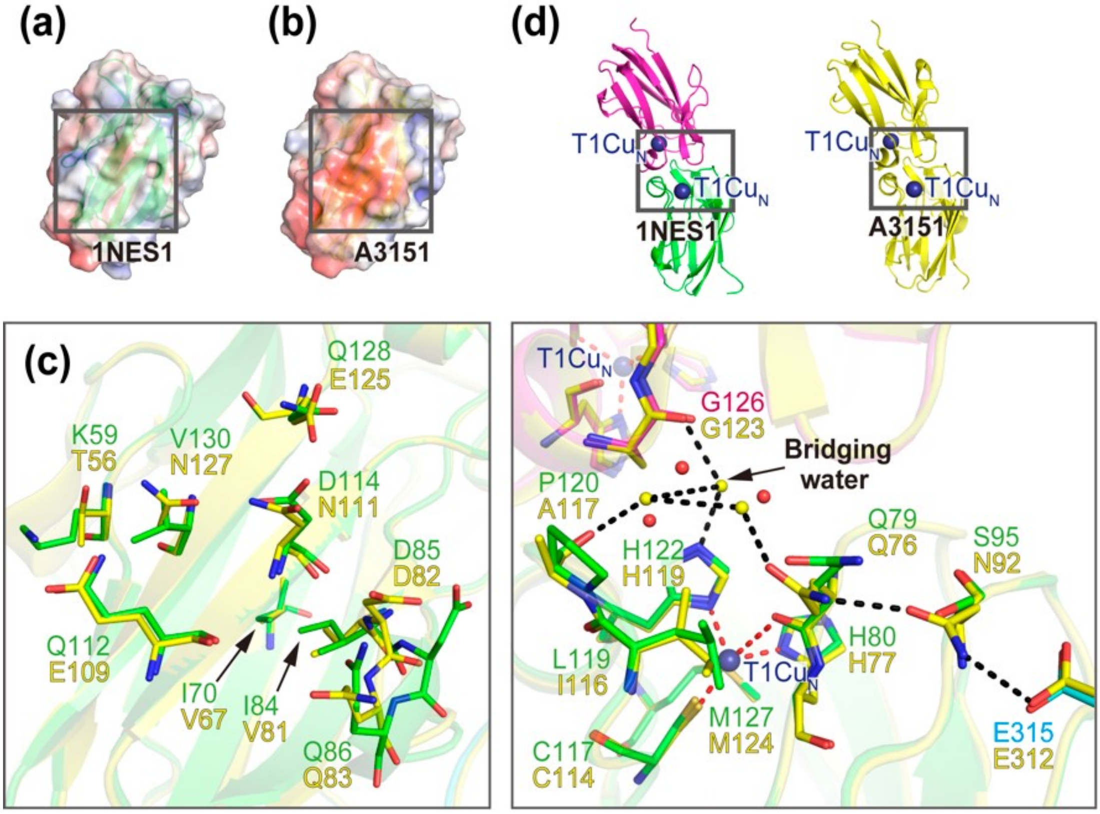

Figure S3 **Structural differences in the extra cupredoxin domain between Hd_{1NES1}NiR and Hd_{A3151}NiR.** Surface charge of the extra cupredoxin domain of **(a)** the Hd_{1NES1}NiR and **(b)** the Hd_{A3151}NiR. **(c)** Surface of the extra cupredoxin domain of the Hd_{1NES1}NiR being coloured in green superimposed on the extra cupredoxin domain of the Hd_{A3151}NiR being colored in yellow. **(d)** Inter-monomer interface of the extra cupredoxin domain of the Hd_{1NES1}NiR being coloured

in green and magenta for each monomer superimposed on the extra cupredoxin domain of the Hd_{A3151}NiR being colored in yellow for all monomer for simplicity. The T1Cu$_N$ ion in extra cupredoxin domain is shown by deep blue sphere. The water molecules for the Hd_{1NES1}NiR and Hd_{A3151}NiR are shown by red and yellow spheres, respectively. The bridging water is indicated by black arrow. Coordination to the T1Cu ion is shown by red broken line. Interaction is shown by black broken line.

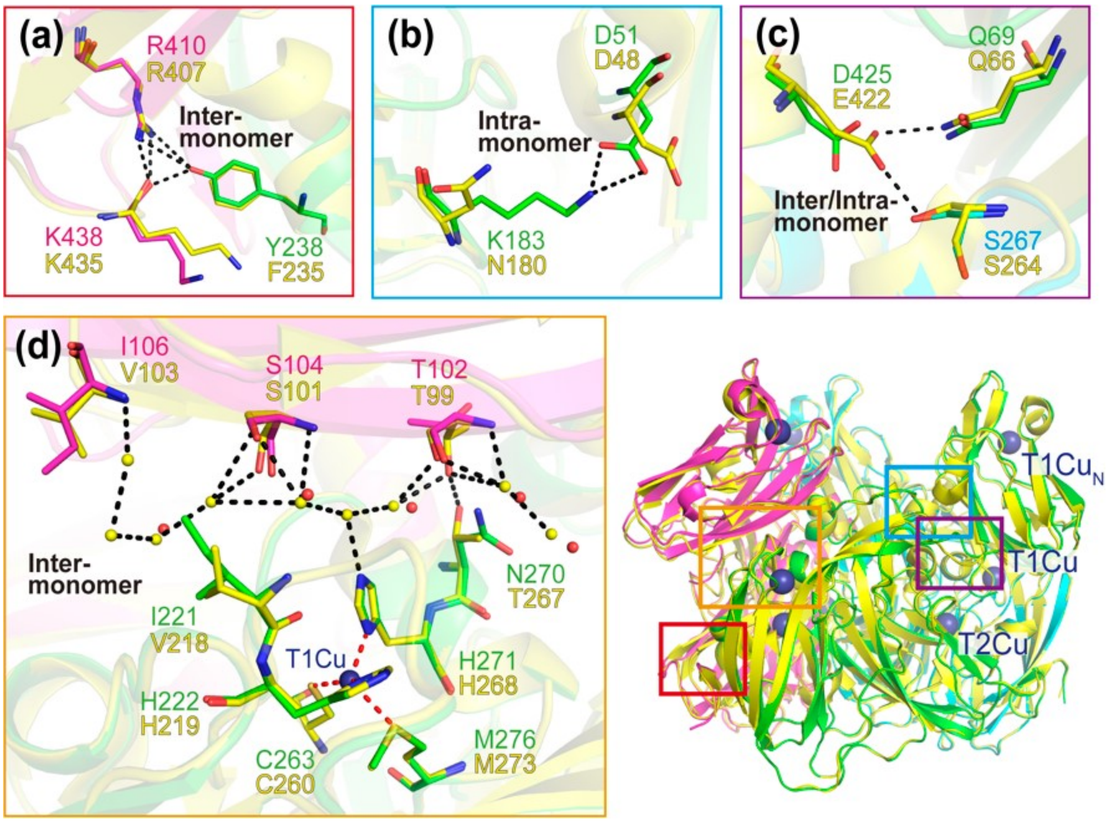

Figure S4 Structural differences in the core domain between *Hd_{1NES1}*NiR and *Hd_{A3151}*NiR. (a, d) Inter- (b) intra- and (c) inter/intra-molecular interface of the trimeric Hd_{1NES1}NiR being coloured in green, magenta and cyan for each monomer superimposed on the trimeric Hd_{A3151}NiR being colored in yellow for all monomers for simplicity. The T1Cu and

T2Cu ions in the core domain and T1Cu$_N$ ion in the extra cupredoxin domain are shown by deep blue spheres. The water molecules for the *Hd*$_{1NES1}$NiR and *Hd*$_{A3151}$NiR are shown by red and yellow spheres, respectively, in (d). Coordination to the T1Cu ion is shown by red broken line in (d). Interaction is shown by black broken line.

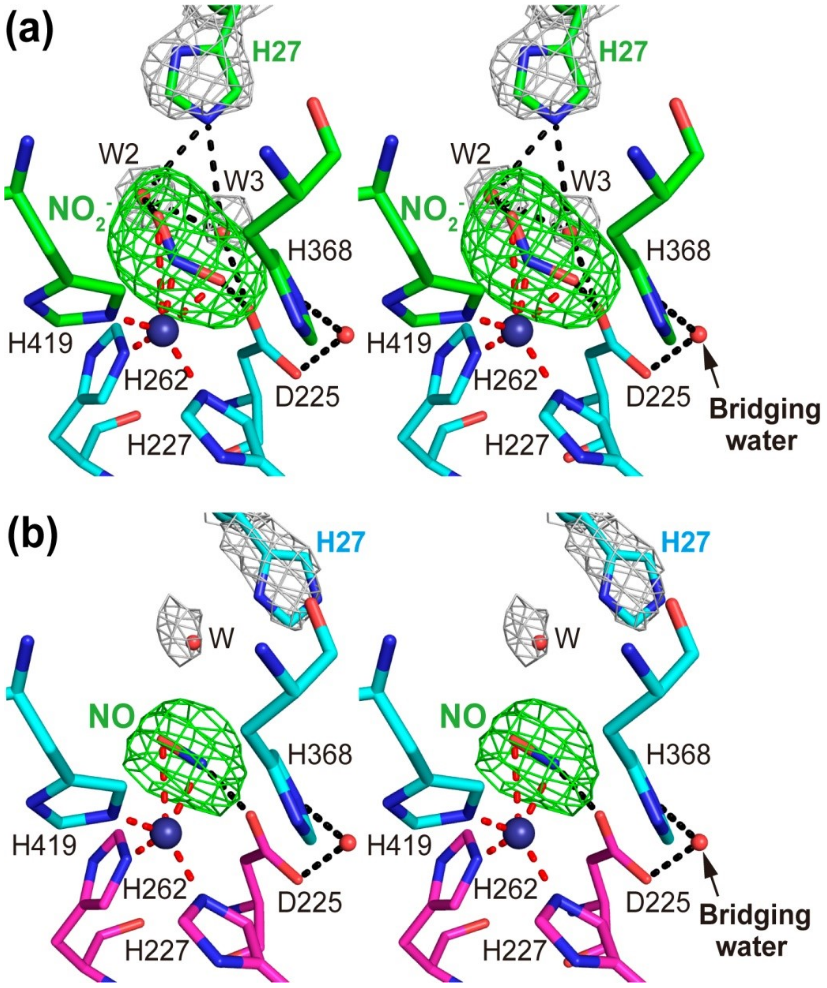

Figure S5 Stereo figures of nitrite and nitric oxide bound to the T2Cu ion of the *Hd*$_{1NES1}$NiR. (a) nitrite (NO_2^-)- and **(b)** nitric oxide (NO)-bound T2Cu of the *Hd*$_{1NES1}$NiR being coloured in green, magenta and cyan for each monomer. The T2Cu ion is shown by deep blue sphere. The water molecules are shown by red spheres. The bridging water is indicated by black

arrow. Coordination to the T2Cu ion is shown by red broken line. Interaction is shown by black broken line. The F_oF_c electron density map at 5.0 σ level is shown for nitrite (NO_2^-) and nitric oxide (NO). The $2F_oF_c$ electron density map at 1.0 σ level is shown for the His27 and the other waters (W2, W3, W).
